# Scanning electron microscope studies of human metaphase chromosomes

**DOI:** 10.1098/rsta.2013.0144

**Published:** 2014-03-06

**Authors:** L. A. Shemilt, A. K. C. Estandarte, M. Yusuf, I. K. Robinson

**Affiliations:** 1 London Centre for Nanotechnology, University College London, 17–19 Gordon Street, London WC1H 0AH, UK; 2 Research Complex at Harwell, Rutherford Appleton Laboratory, Harwell Oxford, Didcot, Oxon OX11 0FA, UK

**Keywords:** scanning electron microscopy, chromosomes, layer structure

## Abstract

Scanning electron microscopy (SEM) is used to evaluate potential chromosome preparations and staining methods for application in high-resolution three-dimensional X-ray imaging. Our starting point is optical fluorescence microscopy, the standard method for chromosomes, which only gives structural detail at the 200 nm scale. In principle, with suitable sample preparation protocols, including contrast enhancing staining, the surface structure of the chromosomes can be viewed at the 1 nm level by SEM. Here, we evaluate a heavy metal nucleic-acid-specific stain, which gives strong contrast in the backscattered electron signal. This study uses SEM to examine chromosomes prepared in different ways to establish a sample preparation protocol for X-rays. Secondary electron and backscattered electron signals are compared to evaluate the effectiveness of platinum-based stains used to enhance the contrast.

## 1. Introduction

X-ray microscopy methods offer a unique application to biology with the ability to produce high-resolution three-dimensional images without sectioning. In recent years, lensless imaging techniques, such as coherent diffraction imaging (CDI) and ptychography, have been used to study biological systems by producing images with resolutions in the 30–50 nm range [1–3]. Sample preparation plays a key role in obtaining the highest resolution possible with these techniques while staying true to the native or natural state. Before doing synchrotron-based X-ray experiments, it is necessary to use other forms of microscopy for testing and optimizing sample preparation.

Chromosomes are dynamic biological objects that undergo large morphological changes during the cell cycle; therefore it is difficult to establish their native state. The process of condensation sees the interphase chromatin become denser as the chromosomes start to form. During metaphase, chromosomes enter several stages of condensation before finally dividing. The great morphological changes during this complex process make it difficult to establish a native state for the chromosomes, and therefore alterations to the structure by sample preparation must be carefully observed so that effects of the protocol are not mistaken for structural features. Fluorescence is used for imaging whole chromosomes; however, it is a low-resolution method that suffers from artefacts when dyes do not faithfully attach to regions of interest. For applications to X-rays, fluorescence microscopy is a useful starting point providing validation of the shape and the successful removal of chromosomes from the nucleus. However, it does not give any information on structures on the length scale to which the X-ray measurements are sensitive. Electron microscopy (EM) can be used in conjunction with fluorescence to provide high-resolution structural information about the chromosome.

EM has several limitations when applied to imaging chromosome structure. In the case of transmission electron microscopy (TEM), the size of the chromosomes exceeds the penetration depth of the electrons, and therefore it cannot be viewed as a whole object. In order to be studied by TEM, chromosomes must go through a sectioning procedure, which disrupts the internal structure, especially the winding of the DNA structure, which is of the most interest. Scanning electron microscopy (SEM) can be used to look at chromosomes in their entirety; however, being a surface-sensitive technique, very little information about the internal structure can be observed.

There have been many previous attempts to image chromosomes with SEM, which have mainly been concerned with their gross morphology [4] and structural changes through mitosis [5]. These works also show evidence of a structural subunit of ‘globular’ form on the surface of the chromosome. This has also been observed in atomic force microscopy (AFM) studies [6]. Sample preparation has a critical effect on the presence and the shape of these globular substructures. Studies show evidence that its shape and size change with sample preparation conditions [7,8]. The effect of different buffers used in sample preparation on the globular structure has been studied with SEM by Sone *et al.* [9]. They observed that surface structure was different between chromosomes prepared in a polyamine buffer and those prepared in a citric acid buffer. For the citric acid preparation, the surface was composed of globular chains 50–70 nm in diameter, whereas polyamine-prepared chromosomes had a flat ‘scaly’ surface with subunits of different sizes. A surface structure has been noted in work with cryo-EM and small-angle X-ray scattering (SAXS). SAXS profiles of chromosomes prepared with typical isolation methods showed a peak at 30 nm. However, chromosomes with the ribosomes removed showed no peaks at this level of structure [10]. This suggests that the ribosomes aggregated to the surface of the chromosome are responsible for the 30 nm structure in chromosomes.

Another key step in sample preparation for SEM is chemical, freeze-drying or critical point drying, because chromosomes are often viewed in vacuum and therefore cannot be imaged in a wet state. It has been found to date, in our experience and by others [4,5], that the globular structure cannot be detected in chromosomes that have been air-dried; therefore, this suggests that drying in this way causes a loss of the surface structural features. Careful drying, such as critical point drying, can be used to slowly dry the chromosome and preserve the surface structures.

Insulating samples build up charge in the SEM, causing artefacts in the image; these can be reduced by making the sample surface conductive. This is usually achieved by coating the sample in a layer of carbon or metal a few nanometres thick. Osmium impregnation has been used in many studies both to improve conductance and to enhance contrast [4,9]. However, it was found that the application of osmium impregnation produces swelling in the chromosome, disrupting the fine surface structure [11]. A sample preparation developed by Wanner and Formanek preserves the surface structure of the chromosome and stains with a contrast-enhancing dye, avoiding toxic osmium impregnation [12]. The stain used in this protocol is a polymer of platinum(II)bis(acetamide) complex [13], which binds to the minor groove of DNA. The platinum ions provide strong contrast in the backscattered electron (BSE) signal, which allows the winding structure of the DNA to be seen in the BSE images. This type of stain has an advantage over osmium-based stains, because it binds only to the DNA and not to the proteins [14]. Wanner & Formanek [15] were able to propose a new structural model of the chromosome using this protocol from barley chromosomes. By imaging chromosomes in different states of condensation, it was observed that the structure was composed of ‘chromomere’ loops, 200 nm in diameter, attached to linear matrix proteins. During the condensation of the chromosomes, the chromomere loops attach to the matrix of parallel fibres, which contract upon condensation.

This protocol has not been successfully applied to mammalian chromosomes because of a mesh-like layer over the chromosomes, which is not present in plants, making barley an ideal subject to study chromosome structure with SEM. In studies by Schroeder-Reiter, this nucleoplasmic layer was digested away with certain proteases; however, it is likely that the surface structure of the chromosome is damaged or modified by this treatment [16]. This layer was also reported by Gautier *et al.* [17], who studied ultra-thin sections of human chromosome spreads with TEM. They observed that this layer was present between chromosomes and had chains of a ‘granular-type structure’.

SEM remains a useful tool to look at the effects of sample preparation on chromosomes owing to its high-resolution imaging of changes in surface structure and the ability to look at the uptake of metal-based stains. This is especially important for X-ray imaging, where the application of heavy metals will increase the scattering power of the object and therefore increase the obtainable resolution. The first three-dimensional image of a chromosome was obtained with CDI at a resolution of 120 nm by Nishino *et al.* [18]. While three-dimensional information was present, this resolution is not sufficient to see the inner structural detail of interest. The chromosomes studied here were unstained and comparison with fluorescence microscopy images was used to analyse structure seen in the X-ray images.

In this study, human metaphase chromosomes are prepared using the ‘drop-cryo’ method and stained with nucleic-acid-specific, contrast-enhancing platinum-based stains, in an aim to understand the applications of this protocol to human chromosome samples. In one set of preparations, Cytoclear, a commercially available mixture of proteases, lipases and detergents, was added to the cell culture step to try and remove the mesh-like layer observed in human chromosomes. The surface structure of the Cytoclear-treated chromosomes was compared to preparations that were obtained from standard cell cultures. Secondary electron (SE) images are analysed to look at the effects of sample preparation. BSE images are analysed to obtain an estimate of uptake of dyes by the DNA. The suitability of this sample preparation protocol for X-ray microscopy methods is discussed. Calculations of estimated phase shift are made from the images obtained in order to provide an estimation of the suitability of ptychography as an imaging method for chromosomes.

## 2. Material and methods

### (a) Chromosome preparation

Chromosomes were prepared from b-lymphocyte cells from a Yoruba cell line (GM18507) which were at passage 4 following a protocol described in [19]. Briefly, the cells were cultured in RPMI-1640 medium (Sigma Aldrich, UK) supplemented with 20% fetal bovine serum (FBS) (Sigma Aldrich) and 1% l-glutamine at 37°C in a 5% CO_2_ incubator. The cells were treated with Colcemid (Gibco BRL) at a final concentration of 0.2 μg ml^−1^. After hypotonic treatment using 0.075 M KCl at 37°C for 5 min, the sample was fixed in three changes of 3 : 1 methanol : acetic acid. One set of chromosomes was prepared with Cytoclear from Genial Genetics (product no. GGS-JL004). This product is designed to remove cytoplasm during cell culture. Here, it was applied during the methanol–acetic acid fixation stage to remove the mesh-like layer. The chromosomes were spread on marked glass slides using the ‘drop-cryo’ protocol established by Wanner *et al.* [12]. The chromosome suspension was dropped onto a slide; afterwards three drops of 45% acetic acid were put on to the slide and a coverslip was placed on top. The slide was then placed coverslip side down on dry ice for 15 min. The spreads were fixed in 2.5% glutaraldehyde in 75 mM cacodylate buffer for 15 min and then washed in 0.1 M cacodylate buffer. The chromosomes were stained with a platinum-based dye, chloro(2,2′:6′,2′′-terpyridine)platinum(II) chloride dihydrate from Sigma Aldrich (catalogue no. 26420), of 10 mM concentration for 15 min. After staining, the preparations were washed in water for 5, 10 and 15 min. The slides were taken through a dehydration series of ethanol, 5 min in 70% solution, 10 min in 85% solution and 15 min in 100% ethanol. Finally the chromosomes were chemically dried using hexamethyldisilazane (HMDS) from Sigma Aldrich, which has been shown to have a similar effect as critical point drying [20].

### (b) Imaging with scanning electron microscopy

The slides were cut and mounted on 12.5 mm stubs. A 3–5 nm layer of carbon was evaporated onto the surface of the stubs. Images were taken with a JEOL Field Emission Gun SEM, model no. JSM-7401F, at 1 kV accelerating voltage. SE images were taken using the gentle beam mode settings, with 3.6 mm working distance. BSE images were obtained at an accelerating voltage of 3 kV at a working distance of 3.1 mm. The globule size was measured from the images by the following analysis. The images were first treated with a Gaussian filter to improve the sharpness over the edges of the globules. A threshold was applied to obtain just the contribution of the globules by removing the background. Measurements of the diameter of the globules were made from these thresholded images with ImageJ. This analysis was performed over different regions of interest in the image. Data from these regions were then combined to produce a histogram of globule size.

### (c) Estimation of scattering performance of stained and unstained DNA

In a coherent diffraction imaging experiment, a diffraction pattern is measured from a coherently illuminated sample. In measuring the intensity of the diffraction pattern, the phase information is lost but can be retrieved using computed algorithms and an image can be inverted from the diffraction data. In CDI, the spatial extent of the beam provides the necessary constraint on the phase retrieval algorithms; therefore it cannot be used for imaging extended samples. Ptychography is an extension of CDI that uses overlapping diffraction patterns to provide a constraint and therefore allows for extended objects to be imaged. From these algorithms, a quantitative measure of amplitude and phase shift through the object is obtained. Phase contrast imaging is the most promising aspect of CDI methods because the phase information obtained by measuring the relative phase shift through an object provides quantitative information about its composition. We can therefore use the information from the SEM about the sample preparation to make estimates of this phase shift.

The relative phase shift Δ*φ* is defined by the following:
2.1
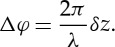


Here *λ* is the wavelength of the incoming X-rays, *δ* is related to the refractive index by *δ*=1−*n*, assuming no absorption, and *z* is the thickness of the material. Since we do not know the sample thickness, we can obtain it indirectly from the known mass of the chromosomes and their area, which can be measured from the SEM images. Thickness is related to area *A* by
2.2
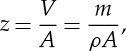
where *V* is the volume, *ρ* is the density and *m* is the mass of the chromosome. We have now introduced the variables of mass and density, which cannot be obtained from the images. The mass of the DNA in a chromosome can be obtained from its genome sequence length and the average molecular weight of 650 amu per base pair. The density of the chromosomes does not have to be estimated, because it is a constant factor in the calculation of the real part of the refractive index *δ*. The value of *δ* can be found for an arbitrary value of density that will then be cancelled in the final calculation of phase shift using the following expression from equations (2.1) and (2.2):
2.3
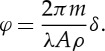


The area of the chromosome was measured from the images using the Measure tool in ImageJ. The mass of this chromosome was estimated from the number of sequenced base pairs of a chromosome of its size. Chromosomes are labelled by size, chromosome 1 being the largest and chromosome Y being the smallest. Compared to the others in the spread, the chromosome was medium-sized so would typically contain 140 Mbp.^1^ This is a big underestimate of the mass of the chromosome, because there are non-transcribing parts of the DNA and proteins that contribute to the mass. However, the number of base pairs per chromosome provides an exact quantification of mass and can be used as a starting point.

For stained chromosomes, the dye was modelled by platinum ions bound to the DNA. The mass of platinum inside the chromosome was estimated by the product of the number of base pairs multiplied by the binding ratio of the dye at 1 platinum ion per 2.5 base pairs [21]. The phase shift was calculated for stained and unstained chromosomes using figure 3*c* for the area measurement. Phase shift from platinum-stained chromosome with a 1 : 1 binding ratio is also calculated to provide a comparison.

## 3. Results

**Figure 1.**
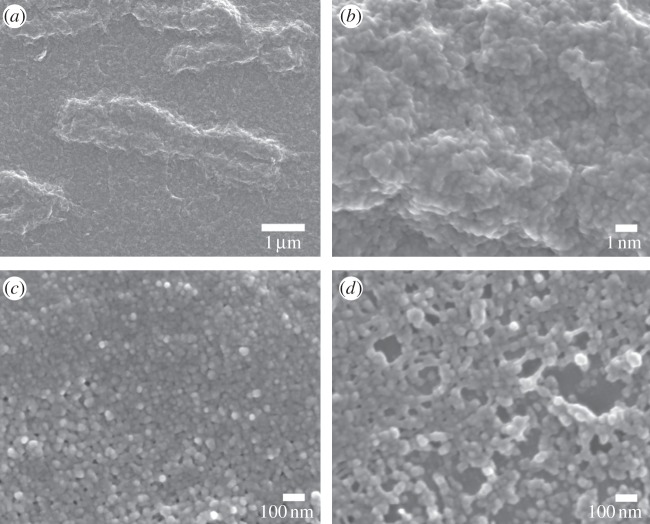
(*a*) SE image of a chromosome prepared without Cytoclear and the layer surrounding it. (*b*) SE image of chromosome in (*a*) at 80 000× magnification. (*c*) SE image of a chromosome prepared with Cytoclear at 80 000× magnification. (*d*) SE image of the mesh layer around the chromosome in (*c*) at 80 000× magnification.

SE and BSE images are used to identify the changes of structure by each step of the sample preparation. The effect of the application of Cytoclear on the chromosome surface structure is shown in figure 1. Preparations without Cytoclear show a heavy mesh layer over the main body of the chromosome (figure 1*a*). The high-magnification image of this chromosome (figure 1*b*) shows that this layer comprises a densely packed globular subunit. Preparations with Cytoclear (figure 1*c*) show a more densely packed globular substructure over the main body of the chromosome. This tightly packed globular structure is not seen in the mesh-like layer between the chromosomes (figure 1*d*); here the globules are attached by fibres. The chemical drying step, with HMDS, has preserved the globular substructure in all cases.

**Figure 2.**
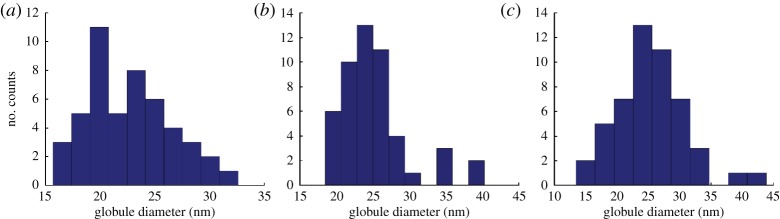
Histograms of the globular subunit diameter (*a*) of a chromosome prepared without Cytoclear, (*b*) in a chromosome prepared with Cytoclear and (*c*) in the mesh layer surrounding the chromosome prepared with Cytoclear. (Online version in colour.)

The size of this globular substructure was measured for the main body of the chromosome from the high-magnification images (figure 1*b*,*c*) and the mesh in between the chromosomes (figure 1*d*). The distribution of diameters is shown in figure 2*a* for the chromosome body prepared without Cytoclear in figure 2*b* for the chromosome body prepared with Cytoclear and in figure 2*c* for the mesh between the chromosomes. The range of diameters of the globular unit is 15–30 nm in all cases, with peaks at 25 nm for the Cytoclear preparation and 20 nm for the preparation without Cytoclear. This suggests that the addition of Cytoclear alters the globular substructure, increasing the diameter.

**Figure 3.**
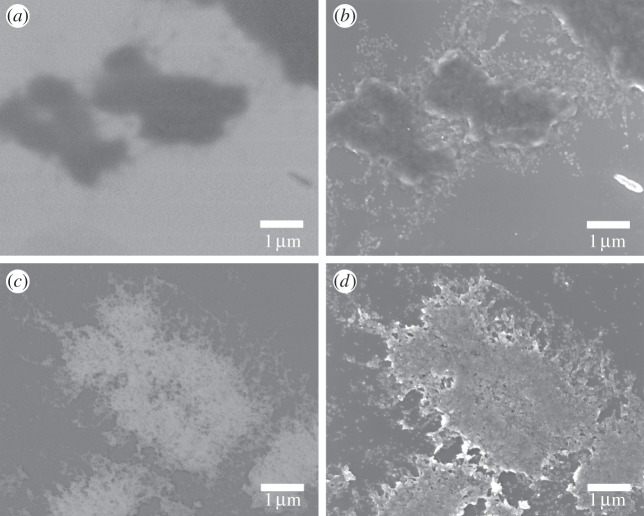
(*a*) BSE and (*b*) SE images of unstained human chromosomes. (*c*) BSE and (*d*) SE images of human chromosomes stained with platinum(II)bis(acetamide) complex.

The consequences of the staining protocol can be observed in the BSE signal (figure 3). The heavy metal stain binds to the DNA and gives some enhancement to the SE images, but the greatest gain in signal contrast is in the BSE images. Like X-ray scattering, the BSE signal increases with atomic number; therefore it is a useful diagnostic for looking at the potential contrast enhancement of the stains when applied to X-ray imaging. As seen in [15], platinum blue is designed to increase the BSE signal from the chromosomes and therefore give information about the structure below the surface level. BSE images of stained and unstained human metaphase chromosomes are shown in figure 3*b*,*d*. There is a large BSE signal from the stained chromosomes, whereas the unstained chromosomes give very little signal, showing that most of the information is coming from the platinum ions within the dye rather than from the organic material of the chromosome itself. This is an important consideration for X-ray sample preparation, as it shows that the signal provided from a stained chromosome is mostly sensitive to the platinum in the stain and not to the structure of the DNA itself. It is, therefore, essential to understand the staining mechanisms of these compounds to the DNA. The scattering from stained objects is subject to artefacts from the unfaithful representation of structure where stains have not attached to the molecules; this is a problem also encountered in fluorescence microscopy.

**Figure 4.**
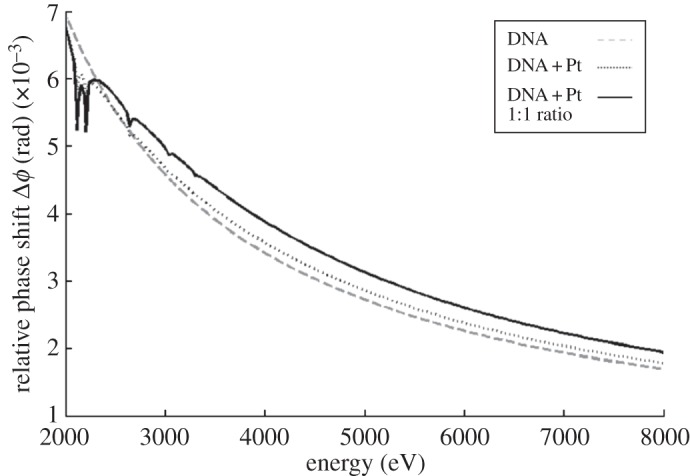
Phase shift from the chromosome in figure 3*c* unstained (dashed) and stained with platinum atoms based on the binding ratio of the DNA-specific stain chloro(2,2′:6′,2′′-terpyridine)platinum(II) chloride (dotted). For comparison, a stain with a binding ratio of one platinum ion per base pair is plotted (solid). Calculated with the CXRO online tool (X-ray optical constants data from http://henke.lbl.gov/optical_constants/) [22].

The potential gain in contrast in X-ray images from these staining methods can be directly calculated from the BSE images. Images produced by X-ray ptychography will give a quantitative measure of the phase shift through the object. The contrast in the images will increase with an increase in phase shift that can be produced by the introduction of heavy metal stains into the chromosomes, much like in the case of BSE. The relative phase shift from unstained and stained DNA was calculated using the area of the chromosome measured from figure 3*c*. The area used in the calculation was 12.3 μm^2^. The relative phase shift was also calculated for a binding ratio of one platinum atom per base pair as a comparison. The relative phase shifts were calculated for the typical energy range used in lensless X-ray microscopy methods, 2–8 keV (figure 4). Biological samples have been measured with ptychography at energies in the range 5–9 keV [23,24].

The relative phase shift is plotted against energy, in figure 4, for the following cases: unstained DNA (dashed line), DNA stained with chloro(2,2′:6′,2′′-terpyridine)platinum(II) chloride (dotted line) and DNA stained with a binding ratio of one platinum ion per base pair (solid line). At low energies between 2 and 3 keV, the relative phase shift is 7 mrad, reducing to around 2 mrad at high energies. There is a steep decline in relative phase shift between 2 and 4 keV, whereas the change in relative phase shift between 6 and 8 keV is much less. Around 2 keV, the unstained chromosomes give greater phase shift than the stained chromosomes because of the absorption of the X-rays by platinum at these energies. Sharp drops in the phase shift of the stained cases can be seen at 2.1–2.2 keV resulting from this absorption. Smaller dips can be seen in the 1 : 1 binding ratio curve at 2.6, 3.0 and 3.2 keV, also corresponding to absorption from the platinum at these energies. The X-ray phase shifts that would be produced by this chromosome is a few milliradians, which is close to but less than the current detection limit for X-ray ptychography [25].

For energies higher than 3 keV, the DNA stained with chloro(2,2′:6′,2′′-terpyridine)platinum(II) chloride gives a marginally greater phase shift than the unstained case. The maximum difference in phase shift between the unstained (dashed line) and stained DNA (dotted line) occurs between 4 and 5 keV and is 0.2 mrad. The difference between unstained DNA and DNA stained with 1 : 1 binding ratio (solid line) of platinum to base pair is 0.5 mrad. At higher energies, the gain in phase shift from chloro(2,2′:6′,2′′-terpyridine)platinum(II) chloride decreases, becoming 0.1 mrad between the stained and unstained cases at 8 keV. The difference between the unstained and the 1 : 1 binding ratio at this energy is 0.2 mrad.

## 4. Discussion

The suitability of the sample preparation protocol for X-ray experiments was investigated with SEM. This type of microscopy provides nanometre resolution information on structural changes and the uptake of heavy metal stains, information that cannot be obtained with fluorescence or light microscopy. Chromosome sample preparations were adapted from SEM protocols for X-ray studies, because there are similar considerations in both methods, such as preventing radiation damage and drying samples, avoiding the evaporation of water, to preserve three-dimensional structure.

The addition of Cytoclear in the sample preparation did not remove the mesh-like layer. In all the examples studied, the globular structure was observed both in the layer and on the surface of the chromosomes. Globule size was found to be between 15 and 30 nm, with a characteristic diameter of around 20 nm, which does not correspond to any of the known structural units of a chromosome, such as the nucleosome (11 nm) and the chromomere (200 nm). However, the measured size of the globules does correspond to the 30 nm ribosome size observed by SAXS [10]. It was observed that the globular subunits are more closely packed in chromosomes that had undergone treatment with Cytoclear and in that case had a larger diameter of 25 nm.

In the measurement of globule diameter, the thresholding of images was affected by the difference in intensity over the region of interest. SEM is a useful microscopy technique, because it has a large depth of field from which surface topography can be seen as a change in greyscale in the image. In the analysis of the images, regions of interest with a small greyscale difference were studied to try and ensure a consistent segmentation. The range of diameters measured was 15–30 nm. Diameters above 30 nm are contributions of two globules that were not separated by the thresholding. A more accurate estimation of globular diameter could be found by employing segmentation techniques. The evaporated carbon layer (3–5 nm thick) will contribute to the measurement of the diameter, leading to an overestimation of diameter size.

The BSE images (figure 3) show that the platinum stain greatly increases the signal contrast. Internal structures and gaps can be seen in figure 3*c* indicating that the dye binds to a fibrous material in the chromosome. Applied to X-rays, this protocol could be used to increase signal, as the uptake of heavy metal dyes by the DNA will increase the electron density in the main body of the chromosome. The mesh-like layer surrounding the chromosomes also stains with platinum; therefore this structure would also be visible in X-ray images and may not be easily distinguishable from the main chromosome structure.

The contribution of staining to X-ray signal is further investigated in the calculation of the relative phase shift. The resulting phase shifts calculated in this way are of the order of a few milliradians, just below the detection limit of ptychography. The behaviour of relative phase shift with energy and staining can provide useful information on the amplification of signal with stain and on the optimal energy to measure chromosomes. The maximum phase shift from the chromosome is at the lowest energy, 2 keV; however, here the difference between the stained and unstained chromosomes is due to the absorption of X-rays by the platinum. At higher energies, the chloro(2,2′:6′,2′′-terpyridine)platinum(II) chloride stain increases the phase shift given by the chromosomes by the order of 0.2 mrad. A 1 : 1 binding ratio of platinum ion to base pair increases this to 0.5 mrad, further indicating that increasing the concentration of bound platinum will provide a gain in relative phase shift. The maximum gain from the platinum is in the energy range 4–5 keV; at energies higher than this, the difference in relative phase shift between the stained and unstained case decreases. It is best to undertake ptychography measurements on stained chromosomes at energies of 4–5 keV, because the gain in phase shift from the stain is greatest and there is no strong absorption from the platinum. The unstained chromosomes provide sufficient phase shift for measurement at 2 keV but would require vacuum conditions to be implemented.

From equation (2.3), it can be seen that the phase shift will increase if the area of the chromosome is reduced. The numerical value of area for the phase shift calculation was taken from the BSE image in figure 3*c* and was measured to be 12.3 μm^2^. If the area of the chromosome was reduced to 1 μm^2^, this would provide a phase shift of 70 mrad at 2 keV, which is within the range to be measured by ptychography. Reducing the area of the chromosomes can be achieved in the preparation stages by using a polyamine buffer in the sample preparation step, which results in smaller chromosomes than a methanol–acetic acid suspension. This change in sample preparation will modify structure; however, it has been shown by light and electron microscopy that the *in vivo* structure of the chromosomes is preserved with polyamines [26]. Methanol–acetic acid preparation is more widely used than polyamine in other chromosome imaging methods, which provide a base for imaging with X-rays; therefore methanol–acetic acid chromosomes were chosen for this study.

The calculation of phase shift only takes into account the mass of the sequenced DNA in the chromosome; therefore it is likely to underestimate the phase shift because contributions from the proteins and non-transcribing DNA have been omitted. This is good for a feasibility study of ptychography, as it is probable that the chromosomes will give significantly higher phase shift than calculated here.

## 5. Conclusion

The images obtained with SEM and the calculations of phase shift indicate that this protocol could be partially applied to ptychography. The SE images show that this protocol preserves the gross morphology of the chromosomes but the Cytoclear step fails to remove the mesh-like layer. The addition of Cytoclear alters the size of the globular subunit seen on the mesh, suggesting that its application changes chromosome structure; therefore this step will be removed in future preparations.

It has been observed that the platinum-based dyes stain the mesh-like layer, demonstrating that it contains nucleic acid. The layer could be caused by nucleic-acid-containing ribosomes attached to the chromosome surface as discussed in §1. Protocols to remove the ribosomes could be employed, but care must be taken to avoid damaging the surface structure of the chromosomes. Further SEM studies could investigate the surface structure of the chromosome after the removal of the ribosome layer.

Our extension of the ‘drop-cryo’ preparation with additional staining from chloro(2,2′:6′,2′′-terpyridine)platinum(II) chloride gives a greater phase shift than the unstained case provided that the absorption edge of platinum is avoided. The estimation of phase shift is currently too small to be measured with ptychography; however it gives an insight into how the phase shift can be increased by alterations to the sample procedure. Firstly, the area of the chromosome could be reduced by suspension in a polyamine buffer instead of methanol–acetic acid. This would give a greater phase shift. Secondly, the binding ratio of heavy metal stains could be increased towards one platinum ion per base pair by using two stains with different binding mechanisms. The most common mechanisms for nucleic-acid-specific dyes are intercalation, where the molecules bind between base pairs, and minor groove binding. It is possible that two dyes could be used together without displacing each other to increase the concentration of the heavy metal in the chromosome.

Our investigations with SEM suggest that a suitable sample preparation for X-ray ptychography would involve the ‘drop-cryo’ preparation of polyamine chromosomes. A procedure of using two stains with different binding mechanisms would be used to increase the density of heavy metal into the chromosome. Careful drying would still be maintained, as the scattering contrast of DNA and air is much greater than DNA in water. While in X-ray imaging there has been a great effort to image biological objects frozen and wet to maintain their structure, SEM images show that fine structural features in the chromosome are preserved by careful drying. This work indicates that it is possible to gain high-resolution ptychographic images with the appropriate sample preparation protocol.

## Footnotes

1 Ensembl. Developed by the Wellcome Trust Sanger Institute and the European Bioinformatics Institute. From database release 72. http://jun2013.archive.ensembl.org/homo_sapiens/, June 2013.
